# Altered Levels and Isoforms of Tau and Nuclear Membrane Invaginations in Huntington’s Disease

**DOI:** 10.3389/fncel.2019.00574

**Published:** 2020-01-17

**Authors:** Marta Fernández-Nogales, José J. Lucas

**Affiliations:** ^1^Instituto Neurociencias Alicante (CSIC-UMH), San Juan de Alicante, Spain; ^2^Centro de Biología Molecular Severo Ochoa (CBMSO)(CSIC-UAM), Madrid, Spain; ^3^Networking Research Center on Neurodegenerative Diseases (CIBERNED), Instituto de Salud Carlos III, Madrid, Spain

**Keywords:** huntington’s disease, tau, tauopathy, tau nuclear rod (TNR), tau nuclear indentation (TNI)

## Abstract

Since the early reports of neurofibrillary Tau pathology in brains of some Huntington’s disease (HD) patients, mounting evidence of multiple alterations of Tau in HD brain tissue has emerged in recent years. Such Tau alterations range from increased total levels, imbalance of isoforms generated by alternative splicing (increased 4R-/3R-Tau ratio) or by post-translational modifications such as hyperphosphorylation or truncation. Besides, the detection in HD brains of a new Tau histopathological hallmark known as Tau nuclear rods (TNRs) or Tau-positive nuclear indentations (TNIs) led to propose HD as a secondary Tauopathy. After their discovery in HD brains, TNIs have also been reported in hippocampal neurons of early Braak stage AD cases and in frontal and temporal cortical neurons of FTD-MAPT cases due to the intronic IVS10+16 mutation in the Tau gene (MAPT) which results in an increased 4R-/3R-Tau ratio similar to that observed in HD. TNIs are likely pathogenic for contributing to the disturbed nucleocytoplasmic transport observed in HD. A key question is whether correction of any of the mentioned Tau alterations might have positive therapeutic implications for HD. The beneficial effect of decreasing Tau expression in HD mouse models clearly implicates Tau in HD pathogenesis. Such beneficial effect might be exerted by diminishing the excess total levels of Tau or specifically by diminishing the excess 4R-Tau, as well as any of their downstream effects. In any case, since gene silencing drugs are under development to attenuate both Huntingtin (HTT) expression for HD and MAPT expression for FTD-MAPT, it is conceivable that the combined therapy in HD patients might be more effective than HTT silencing alone.

## Introduction

### Huntington’s Disease Overview

Huntington’s disease (HD) is an autosomal dominant neurodegenerative disorder caused by a mutation in the gene that encodes for the protein Huntingtin (Htt). This mutation consists on an abnormal expansion of a CAG triplet (>35) in exon 1 of the gene that encodes for a stretch of polyglutamine (polyQ) in the N-terminal region of the protein (Gusella et al., 1993; MacDonald et al., 1993) and, thus, HD belongs to the group of the polyglutamine diseases (Zoghbi and Orr, 2000). Clinically, HD patients suffer multiple symptoms that include motor (involuntary body movements, chorea, dystonia, and gait abnormalities), cognitive (decreases in attention and mental flexibility) and psychiatric and/or behavioral impairment (apathy, irritability, impulsivity, depression and suicidal wishes) due to the affectation of different parts of the brain (Vonsattel and DiFiglia, 1998; Sturrock and Leavitt, 2010; Kim and Fung, 2014). Population without the disease has between 6 and 35 CAG triplets in the *HTT* gene, while individuals with expansions of 40 or more repeats develop HD. Carriers of 36–39 CAG repeats have lower penetrance and later onset of the disease (Andrew et al., 1993). There is a relationship between the length of CAG repeat and the onset and severity of the disease leading to a worse prognosis as the length increases [Snell et al., 1993; Genetic Modifiers of Huntington’s Disease (GeM-HD) Consortium, 2019].

As in other polyglutaminopathies, illness is mainly due to a toxic gain of function of the expanded polyQ-containing protein and also of the expanded CAG-containing mRNA (Shieh and Bonini, 2011; Nalavade et al., 2013; Martí, 2016) rather than to a loss of function of mutant Htt (mHtt). Although the latter may also contribute to some of the HD-specific symptoms (Zuccato and Cattaneo, 2014). The Htt protein is expressed ubiquitously throughout the body with high levels in the brain and testes (Schulte and Littleton, 2011). It interacts with different partners that are implicated in cellular dynamics -like cytoskeleton, endocytosis, trafficking, and adhesion-, metabolism, protein turnover, transcription and RNA processing (Kaltenbach et al., 2007) and participates in vesicular transport, synaptic transmission and autophagy, playing a role in embryogenesis, signal transduction and cell adhesion (Schulte and Littleton, 2011; Smith-Dijak et al., 2019). The absence of Htt causes embryonic lethality while mice lacking one Htt allele do not show phenotypical changes (Nasir et al., 1995).

Regarding neuropathology, HD is characterized by neuronal death, primarily of medium-sized spiny neurons of the striatum producing a progressive atrophy of the basal ganglia (Hedreen and Folstein, 1995; Mitchell et al., 1999) but also in other structures that are related to cognition such as the cortex and hippocampus (Zheng and Diamond, 2012), which explains the different symptoms that patients suffer. Nowadays, there is no cure for HD and, normally, death takes place between 15 and 20 years after the onset of the symptoms.

Truncated N-terminal portions of mHtt can be generated through proteolytic cleavage by caspases, calpains or other endoproteases and this favors the agglomeration of mHtt—driven by the self-aggregation of polyQ. This process eventually leads to the formation of oligomers and globular intermediates that can interfere with multiple intracellular functions in the cytoplasm (such as organelle and mRNA transport, protein turnover, or mitochondrial function among others) and in the nucleus where gene expression can get altered (Graham et al., 2006). Besides, N-terminal fragments of mHtt are not only produced by proteolytic cleavage but also by aberrant splicing (Bates et al., 2015). Recently, it has been observed in a cohort of 56 HD patients a decrease in the levels of total Htt levels (using EM48 and CH00146 antibodies) and an increase in the levels of the N-terminal fragment without alteration in Htt mRNA levels. This is accompanied by an increase in Htt oligomers without changes in monomers according to Sarkosyl based protein fractionation, suggesting that abnormal translation and/or protein turnover is responsible for Htt misregulation in HD (St-Amour et al., 2018).

The aggregation of mHtt produces an histopathological mark in form of spherical inclusions that are detected in the nuclei and cytoplasm of neurons in HD patients (DiFiglia et al., 1997) but also in different transgenic animal models like mice (Davies et al., 1997) or *Drosophila* (Warrick et al., 1998). Inclusions can be detected using antibodies against Htt but also against other epitopes like polyQ or ubiquitin (DiFiglia et al., 1997; Sapp et al., 1997; Vonsattel et al., 2008). There is growing evidence that HD inclusions, in addition to mHtt, can nucleate other proteins like those that are characteristic of other neurodegenerative diseases, like α-synuclein -found in Parkinson’s disease (Corrochano et al., 2012; Herrera and Outeiro, 2012; Tomas-Zapico et al., 2012), TDP-43 -found in amyotrophic lateral sclerosis- (Schwab et al., 2008; Coudert et al., 2019), or Tau -found in Tauopathies such as Alzheimer’s disease (Fernández-Nogales et al., 2014; Vuono et al., 2015; St-Amour et al., 2018).

### Tau Overview

#### Tau Gene and Isoforms Generated by Alternative Splicing

Tau is a microtubule-associated protein (MAP) that was first discovered in 1975 by Weingarten when it was co-purified along with tubulin (Weingarten et al., 1975). In humans, this protein is encoded by the gene *MAPT* that is located in the region q21.31 on chromosome 17 and contains 16 exons (Neve et al., 1986; Andreadis et al., 1992), while in mouse is located on chromosome 11. The human MAPT gene has two haplotypes, the more common H1 and the unusual H2 haplotype. The latter results from a large—approximately 970 kb—chromosomal inversion and a 238 bp deletion in intron 9 (Stefansson et al., 2005; Caillet-Boudin et al., 2015).

The MAPT gene is expressed at its highest levels in neurons in the central nervous system where it contributes to the maintenance of neuronal polarity by promoting microtubule assembly and stability.

Multiple Tau isoforms are generated by alternative splicing. The exons that are alternatively spliced in adult CNS are exons 2, 3 and 10, and their combinatorial usage generates six Tau isoforms in the adult human brain (Andreadis, 2005; Liu and Gong, 2008). On one hand, alternative splicing of exons 2 and 3 generates Tau isoforms that differ by the absence or the presence of an insert of 29 or 58 amino acids—corresponding to exons 2 or 2 and 3—in the N-terminal region. Thus, exon 2 can appear alone but exon 3 never appears independently of exon 2. This way the so-called 0N, 1N or 2N isoforms are generated (Andreadis et al., 1995). On the other hand, the exclusion or inclusion of exon 10—encoding a 31 amino acid sequence that provides one of the four possible tubulin-binding repeats in the C-terminal region of the protein—results in either 3R or 4R isoforms (Goedert and Spillantini, 2011).

The N-terminal region binds to plasma membrane components to regulate interactions while the C-terminal part of the protein binds microtubules (Derisbourg et al., 2015). If we focus our attention in the C-terminal region, 4R-Tau isoforms bind microtubules with higher affinity and are more efficient at promoting microtubule assembly *in vitro* compared to 3R-Tau isoforms that have less affinity for microtubules (Lu and Kosik, 2001). More precisely, both 4R-Tau and 3R-Tau isoforms increase the growth rate of microtubules, but 3R-Tau shows less efficacy in protecting microtubules from disassembly than 4R-Tau isoforms (Panda et al., 2003). During development, 0N3R isoform is the most abundant making 3R-Tau to predominate over 4R-Tau (Kosik et al., 1989). However, in healthy human adult brain, 3R-Tau and 4R-Tau are equally represented (Goedert et al., 1989). In the case of the adult mouse brain, 4R-Tau isoforms are predominant (Kosik et al., 1989; Takuma et al., 2003). The microtubule-binding repeats also comprise the paired-helical filament (PHF) core that is the primary structure of aggregated Tau filaments (Wischik et al., 1988) and dysregulation of the balance between 3R-Tau and 4R-Tau isoforms has been shown to contribute to neurodegeneration (Liu and Gong, 2008), as we are going to comment in more detail.

#### Functions of Tau

In healthy neurons, Tau is almost exclusively located in the axon (Wang and Mandelkow, 2016) where, as mentioned, it is implicated in microtubule assembly and stabilization. Microtubules are polar structures with a plus and a minus-end and they are formed from α and β-tubulin heterodimers. Tau binds both α and β-tubulin subunits and can promote microtubule growth (Witman et al., 1976; Kadavath et al., 2015). The assembly of microtubules consists of a phase of rapid polymerization and a steady-state, where no assembly occurs. Assembly occurs at the plus end while disassembly occurs at the minus end, keeping the overall length of the microtubule equal. Tau reduces the frequency of depolymerization by binding along the outer surface. Microtubule dynamics in the nervous system requires a high degree of stability. While the N-terminal region of Tau could contribute to the formation of microtubule bundles as it functions as a spacer in between them (Chen et al., 1992), the C-terminal region binds to microtubules to regulate their polymerization (Cleveland et al., 1977). In healthy conditions, due to its ability to modify microtubule dynamics (Trinczek et al., 1999; Dixit et al., 2008), Tau contributes to regulate different cellular functions such as transport of mRNA and proteins along the axons, as well as neurite extension. Accordingly, when Tau is knocked down, neurite formation is inhibited altering processes such as neuronal differentiation or synaptic plasticity (Caceres and Kosik, 1990; Kempf et al., 1996; Stamer et al., 2002; Spillantini and Goedert, 2013). Moreover, Tau is present in small amounts in dendrites and even in the nucleus where it can bind to DNA protecting it from damage (Wei et al., 2008; Violet et al., 2014). DNA binding of Tau takes place at both genic and intergenic regions (Benhelli-Mokrani et al., 2018) and results in modulation of gene expression (Siano et al., 2019). Interestingly, nuclear Tau has also been involved in nucleolar transcription (Maina et al., 2018) and the decrease in nuclear Tau (detected with Tau-100 antibody) that takes place in AD CA1 and dentate gyrus neurons along disease progression might be pathogenic by leading to decreased protein synthesis (Hernández-Ortega et al., 2016). Finally, different mutations that alter the proportion of Tau isoforms as well as post-translational modifications can modify the affinity of Tau for microtubules. The differential interaction of 4R-Tau and 3R-Tau with the microtubules may have important implications for neuronal diseases as the regulated expression of both isoforms is required for the correct function of the neurons. Therefore, in summary, both a loss of function of Tau and a toxic gain of function due to aggregate formation can contribute to neurodegeneration.

#### Post-translational Modifications of Tau

Tau protein can be modified after translation by phosphorylation, glycosylation, ubiquitination, acetylation or truncation, among others (Martin et al., 2011). Regarding phosphorylation of Tau, it can be phosphorylated in serine, threonine and tyrosine residues with 85 potential sites of phosphorylation, of which 45 have been validated (Wang and Mandelkow, 2016). The sites of phosphorylation can be divided depending on the kinases that can phosphorylate them: proline-directed kinases -like glycogen synthase kinase 3 (GSK-3), cyclin-dependent kinase 5 (CDK-5), cyclin-dependent kinase 1 (CDK-1), mitogen-activated protein kinase (p38), c-Jun N-terminal kinases (JNK), and other stress kinases—and non-proline-directed kinases—like protein kinase A (PKA), protein kinase C (PKC), calmodulin kinase II (CaMK-II), microtubule affinity regulating kinase (MARK) or casein kinase 2 (CK2).

Phosphorylation of Tau reduces its affinity for microtubule and different plasma membrane components, thus reducing microtubule stability. For example, phosphorylation on Ser-214 and Thr-231 promotes detaching of Tau from microtubules. Deregulation of Tau phosphorylation is deleterious for neurons and has been implicated in many diseases such as AD, where Tau hyperphosphorylation favors its detachment from the microtubules thus increasing the levels of soluble Tau available for self-aggregation leading to the formation of neurofibrillary tangles (NFT) and/or neuropil threads (NT; Wang and Mandelkow, 2016). GSK-3 is believed to play an important role in Tau hyperphosphorylation as it is able to phosphorylate the majority of the residues which are hyperphosphorylated in AD (Lovestone et al., 1994) and its levels are increased in AD brains (Pei et al., 1997). Apart from kinase hyperactivity, dysregulation of phosphatases can also lead to pathogenic hyperphosphorylation. Different phosphatases such as PP1, PP2A, PP2B (calcineurin) and PP2C can eliminate phosphates from Tau but only PP1, PP2A, and PP2B (Gong et al., 2004) have been shown to dephosphorylate abnormally hyperphosphorylated Tau.

Not only phosphorylation modulates Tau activity. Acetylation can also regulate its function as it has been observed that, *in vitro*, it can preclude microtubule assembly (Min et al., 2010; Cohen et al., 2011). Important sites of acetylation are K163, K274, K280, K281, and K369, with K281 and K274 being acetylated in AD patients (Tracy et al., 2016). Interestingly, when the levels of acetylated Tau are increased, its levels of phosphorylation are reduced (Min et al., 2010).

Other post-translational modifications that include N-glycosylation, truncation and isomerization stabilize PHFs. It has been described that N-glycosylation is related to Tau hyperphosphorylation and Tau aggregation (Ledesma et al., 1995). N-glycosylation stabilizes aggregated PHFs leading to tangle formation in AD. Phosphorylation on Ser-717 completely abolishes the O-GlcNAcylation on this site, while phosphorylation on Ser-713 and Ser-721 reduces O-GlcNAcylation. O-GlcNAcylation on Ser-717 decreases the phosphorylation on Ser-721 by about 41.5%. Truncation of Tau can also promote aggregation as Tau fragments have been found in the PHFs of AD patients (Wischik et al., 1988).

Finally, Tau can be ubiquitinated and this modification has mainly been found in aberrant aggregates such as the inclusion bodies found in Pick’s or Parkinson’s diseases or in PHFs in AD (Mayer et al., 1989; Morishima-Kawashima et al., 1993). PHF-Tau can be modified by three different forms of poly-ubiquitination, “Lys-48”-linked poly-ubiquitination is the major form but “Lys-6”-linked and “Lys-11”-linked poly-ubiquitination could also occur.

#### Tau Mutations and Tauopathies

The alteration of the amount and the structure of the Tau protein can disturb its localization and, as a consequence, its function producing different pathological effects. Tauopathies are a class of neurodegenerative disorders that are characterized by the aggregation and intracellular deposition of Tau in neurons and/or glial cells as a consequence of abnormal increase in the levels of phosphorylation, abnormal splicing of the mRNA or mutations in *MAPT* gene.

Tauopathies can be divided into: (a) primary Tauopathies that are a major class of Frontotemporal Lobar Degeneration (FTLD) neuropathology and can present clinically with several forms of Frontotemporal Dementia (FTD)—like Frontotemporal Dementia with parkinsonism linked to chromosome 17—(FTDP-17), progressive supranuclear palsy syndrome (PSP) or corticobasal degeneration (CBD); and (b) secondary or non-primary Tauopathies like Alzheimer’s disease (AD) in which neurofibrillary Tau neuropathology occurs in addition to the amyloid-β (Aβ) plaques. The various Tauopathies affect different brain regions and cell types and they also show differences in the ratio of Tau isoforms present in the Tau filaments (Sergeant et al., 2005). In AD, there are similar levels of 3R-Tau and 4R-Tau in the PHFs (Goedert et al., 1995) while other Tauopathies like PSP or CBC show an increase in 4R-Tau isoforms and others like PiD show an increase in 3R-Tau isoforms (Arendt et al., 2016).

Missense, silent and intronic mutations in the MAPT gene have been directly related to different Tauopathies or constitute a risk factor for them (Goedert and Jakes, 2005). In 1998, it was discovered that mutations on the *MAPT* gene cause FTDP-17, confirming that Tau dysfunction is sufficient to cause neurodegeneration. These patients showed filamentous Tau inclusions in neurons and glia and atrophy of the frontal and temporal lobes (Hutton et al., 1998; Poorkaj et al., 1998; Spillantini et al., 1998). There are different missense mutations like ΔK280, P301L, G272V, V337M, R406W and N279K that reduce microtubule assembly contributing to PHFs stabilization and its aggregation forming NFTs (Barghorn et al., 2000). Regarding intronic mutations, they are located around exon 10 affecting its rate of inclusion and they lead to an imbalance of the ratio of 4R- and 3R-Tau isoforms (Liu and Gong, 2008).

## Tau Pathology in Huntington’s Disease

Along the last decades, many studies have demonstrated Tau alterations and Tau-positive histopathological hallmarks in HD patients as well as in animal models that could be contributing to the progression of the disease. Here, we aim to review these pieces of evidence to elucidate the role of Tau in the disease and to elaborate on whether this offers opportunities for therapeutic interventions to ameliorate HD prognosis.

### Polymorphisms

It has been described that the MAPT H1 haplotype—that is the most abundant—could be a genetic risk factor for some Tauopathies like PSP or CBD (Houlden et al., 2001; Pittman et al., 2004). Also, H1 haplotype increases the expression of total MAPT transcript as well as specifically increases the inclusion of exon 10 and therefore the proportion of 4R-Tau isoforms (Myers et al., 2007). In HD, Tau polymorphisms have been linked to the progression of cognitive deficits. In a group of 960 HD patients that were genotyped for the H1 and H2 haplotypes- using the SNP rs9468, Vuono et al. (2015) reported that there is a correlation between increased number of CAG repeats and increased rate of cognitive decline in H2 carriers and that Tau H2 haplotype carriers show accelerated cognitive deterioration compared to H1/H1 homozygous carriers.

### Tau Levels

Regarding Tau protein levels, a high increase in the levels of total Tau (Tau-5 antibody) was found in the cortex of HD patients and this is accompanied with the appearance of lower molecular weight (35 and 39 KD) bands (Fernández-Nogales et al., 2014), while no changes were found in the striatum (Fernández-Nogales et al., 2014). However, a more recent report has shown elevated Tau total mRNA levels in the putamen of HD patients (St-Amour et al., 2018). Importantly, the presence of an excess of Tau in HD brains most likely contributes to disease as Tau knock-down has been demonstrated to attenuate motor abnormalities in an HD mouse model (Fernández-Nogales et al., 2014).

### Aberrant Splicing of Tau in HD

As previously commented, in some Tauopathies including PSP, CB, Pick’s disease (PiD) and FTLD with Tau+ inclusions (FTLD-Tau), alteration of alternative splicing of exon 10 produces an imbalance in 4R-Tau and 3R-Tau isoforms (Park et al., 2016). Regarding HD, we demonstrated that patients (and mouse models of the disease, like the R6/1 and HD94 mice) show an increase in the ratio 4R-Tau/3R-Tau mRNA isoforms in cortex and striatum, accompanied by an increase of the levels of 4R-Tau protein. In the striatum, there also was a decrease in 3R-Tau protein (Fernández-Nogales et al., 2014). The imbalance of 4R-Tau/3R-Tau mRNA isoforms was corroborated by Vuono et al. (2015) in the cortex and striatum of a cohort of 16 patients. More precisely, they detect 1N3R and 2N4R mRNA and protein isoforms and found an increase in 4R-Tau isoforms that leads to an altered ratio of isoforms. Recently, using putamen samples from a higher number of patients (St-Amour et al., 2018), it was shown a 2.5-fold increase in 4R-Tau/3R-Tau ratio at the protein level and a 1.8-fold increase at the mRNA level, due to an upregulation of 4R-Tau isoforms. The high number of samples analyzed in that study allowed them to conclude that the top increment takes place in grade 2 cases regarding the mRNA and in grade 3 cases regarding the protein, resulting in higher 0N4R and lower 1N3R isoforms (St-Amour et al., 2018). All these findings regarding altered isoform ratio in HD are very relevant in view of the fact that alteration in the ratio of 4R-Tau and 3R-Tau isoforms is sufficient to cause neurodegeneration (Hutton et al., 1998; Qian and Liu, 2014), as this might contribute *per se* to HD neurodegeneration independently of other deleterious effects of mHtt, thus becoming a therapeutic target for HD.

Alternative splicing of exon 10 is regulated by a system of factors that bind to the RNA regulating the splicing of the pre-mRNA itself. Among these factors, the family of the serine- and arginine-rich (SR) proteins participate on constitutive splicing while also regulating alternative splicing. Some members of this family promote the inclusion of exon 10, while others suppress it. Several studies have shown that SRSF1 (ASF/SF2), SRSF2 (SC35), SRSR6 (SRp55), and SRSF9 (SRp30c) promote exon 10 inclusion, while SRSF3, SRSF4, SRSF7, and SRSF11 suppress its inclusion (Qian and Liu, 2014).

It was bioinformatically predicted (Sathasivam et al., 2013) and biochemically confirmed (Schilling et al., 2019) that the splicing factor SRSF6 can bind CAG RNA repeats and this leads to incomplete splicing of Htt RNA and to production of a small form of Htt known as exon 1-Htt (Sathasivam et al., 2013). Besides, SRSF6 was indeed found altered in the striatum of HD patients and in the R6/1 mouse model of the disease, as it gets sequestered into mHtt inclusions (Fernández-Nogales et al., 2014). The activity and localization of SR proteins could be modulated by post-translational modifications such as phosphorylation of their multiple serine and threonine residues, and such phosphorylation is required, in general, for the translocation of SR proteins from the cytoplasm to the nucleus. In this regard, it has been shown an increase in the levels of phosphorylation of SRSF6 in the striatum and cortex of HD patients and R6/1 mice (Fernández-Nogales et al., 2014) which may favor dissociation from nuclear speckles (Yin et al., 2012; Naro and Sette, 2013). This, together with the sequestration of SRSF6 into mHtt inclusions, suggests a decrease in SRSF6 activity that could explain the modulation observed of exon 10 splicing in HD. Moreover, SRSF6 not only modulates alternative splicing of MAPT, as it also modulates alternative splicing of MAP2, another MAP whose alternative splicing is altered in HD (Cabrera and Lucas, 2017).

Recently, it has been proposed the splicing factor proline- and glutamine-rich (SFPQ) could also be responsible for the 4R-Tau/3R-Tau imbalance as it has been shown to modulate exon 10 splicing and to interact with FUS, one major component of mHtt inclusions (Fujioka et al., 2013; Ishigaki et al., 2017). Although no association was apparent between SFPQ nuclear signal intensity and presence or absence of HD-associated intranuclear inclusions in striatal and cortical neurons of seven HD cases (Baskota et al., 2019), the reduced nuclear availability of free FUS in HD and, as a consequence, a decreased interaction with SFPQ, might affect 4R-Tau/3R-Tau ratio.

More recently, St-Amour et al. (2018) have explored the alternative splicing that affects exon 2 and 3 on the MAPT gene. It has been observed an increase of 1.7 fold-change in the isoforms that do not contain exons 2 and 3 (0N-Tau) at mRNA level and a 0.5 fold-change in the isoforms that only contain exon 2 (St-Amour et al., 2018). Further investigation is required to know the effect of the alteration in the ratio of these isoforms in the progression of the disease as the N-terminal region of Tau participates in the interaction with different membrane components.

### Tau Phosphorylation in HD

As previously described, Tau functions are modulated by site-specific phosphorylation and its alteration produce a toxic loss of function as the microtubule-binding ability is decreased, but also a toxic gain of function as it generates deposits as a result of its aggregation. There are multiple studies showing Tau hyperphosphorylation in HD that could contribute to the disease. The first evidence of Tau hyperphosphorylation in HD patients was obtained by immunohistochemistry with the AT8 antibody which revealed positive staining -with or without NTs in 13 of 27 analyzed patients (Jellinger, 1998). In good agreement, a recent study on 16 cases including young HD cases (26 and 40 years) has shown AT-8 positive neuronal inclusions with different shapes and conformations like ring-like perinuclear, flame-shaped and globular inclusions in the cortex and striatum as well as astrocytic plaques, NT, dots and nuclear rods (Gratuze et al., 2015; Vuono et al., 2015). Using antibodies other than AT-8, an increase in Tau phosphorylation at Ser396/Ser404/Ser199 and Thr205 epitopes, while no changes in epitopes Ser235/Ser262/Ser356, has been observed in the putamen of a cohort of 56 patients (St-Amour et al., 2018). Recently, hyperphosphorylated Tau (detected with antibodies AT8, CP13, AT180b and PHF-1) has been detected in fetal tissue transplanted into cortex and striatum of two HD cases (Cisbani et al., 2017).

There is some controversy regarding the presence of hyperphosphorylated Tau in the Sarkosyl-insoluble fraction obtained from HD brains. While Vuono et al. (2015) found elevated AT-8 Tau in this fraction, two different studies failed to replicate this. More precisely, we did not detect hyperphosphorylated Tau (with AT-8 or with PHF-1) in this insoluble fraction of HD brains (Fernández-Nogales et al., 2014) and neither did the more recent study by St-Amour et al. (2018) which analyzed a much higher number of HD cases. Interestingly, in the latter, they report that multiple antibodies against Tau phospho-epitopes were below detection levels in the Sarkosyl-insoluble fraction.

Regarding cellular and animal models of HD, mHtt expression promotes Tau hyperphosphorylation at Ser396 as evidenced in cells in which Tau was co-transfected with mHtt, in contrast to what happens when Tau is co-transfected with wild type Htt in which the levels of phosphorylation are maintained stable (Blum et al., 2015). The Q111 striatal knock-in cellular model that mimics the polyglutamine expansion shows Tau hyperphosphorylation when there is pharmacological inhibition of PP2B/calcineurin in comparison with Q7 cells (Gratuze et al., 2015).

In animal models of HD, there are plenty of demonstrations of Tau hyperphosphorylation. Thus, Gratuze et al. (2015) showed increased Tau hyperphosphorylation at PHF-1 epitope in presymptomatic R6/2 whereas symptomatic R6/2 mice displayed Tau hyperphosphorylation at multiple Tau phospho-epitopes like AT-8, CP13, PT205 and PHF1 in the hippocampus. Besides, the zQ175 knock-in mice show increased phosphorylation with PS199. Similarly, Blum et al. (2015) showed that R6/2 and KI140 mice display increased phosphorylation in Ser404 and Ser396 by western blot and also by immunofluorescence with the pSer396 antibody in KI140 mice. Besides, they also detected a decrease in Tau1 (unphosphorylated Tau) in R6/2 and KI140 mice.

The phosphorylation of Tau is a balance between the activity of its kinases and phosphatases (Sergeant et al., 2008). One of the main kinases that phosphorylates Tau is GSK-3 which has been shown to mediate Tau phosphorylation in Alzheimer’s disease (Hernandez et al., 2013) and bipolar disorder (Beaulieu et al., 2004; Li et al., 2010). However, there is a dramatic decrease in GSK-3 levels and activity in the striatum and cortex from HD patients (Lim et al., 2014; Fernández-Nogales et al., 2015) as well as in R6/1 mouse model (Saavedra et al., 2010; Fernández-Nogales et al., 2015), while an increase in active pGSK-3β-Tyr^216^ does take place in hippocampus of HD patients and R6/2 mice (L’Episcopo et al., 2016). It has been reported that GSK-3β, CamKII, AKT, JNK, p38, ERk or CDK-5 are not activated in R6/2 and Q175 mice and there is even increased Ser9 phosphorylation of GSK-3β (resulting in the inactive form of the kinase) and reduced phosphorylation and levels of CamKII, as well as reduced cdk5 and ERK expression in the cortex of R6/2 and KI140 mice (Deckel et al., 2002; Blum et al., 2015; Gratuze et al., 2015). Together, all these results do not fit with the Tau hyperphosphorylation observed in HD. In contrast, and regarding the phosphatases implicated in Tau dephosphorylation, it has been shown a decrease in PP1, PP2A and PP2B expression in R6/2 mouse model and a significant reduction in Calcineurin/PP2B expression in KI140 (Blum et al., 2015; Gratuze et al., 2015) which may explain the hyperphosphorylation phenotype.

### Tau Truncation in HD

As mentioned, Tau truncation may be relevant to neurodegeneration as it may alter the Tau function and favor the formation of Tau aggregates. There are different truncated forms of Tau depending on the protease responsible for the cleavage. The ΔTau314 has been demonstrated to be generated by Casp2 and to cause synaptic dysfunction and cognitive deficits in cellular and transgenic mouse models of FTDP-17 (Zhang et al., 2014). Recently, it has been reported that both Casp2 and ΔTau314 proteins are higher in the striatum (caudate nucleus) and prefrontal cortex (Brodmann’s area 8/9) of HD patients as compared non-HD individuals (Liu et al., 2019).

## Tau-Positive Nuclear Membrane Invaginations and Other Histopathological Marks in HD

For decades, there have been numerous histopathological reports of HD patients in whom the presence of NFT—the histopathological hallmark characteristic of different Tauopathies such as Alzheimer’s disease—has been detected (McIntosh et al., 1978; Myers et al., 1985; Reyes and Gibbons, 1985; Moss et al., 1988; Caparros-Lefebvre et al., 2009). More systematic studies using larger patient cohorts have detected Tau pathology in 60% (16/27; Jellinger, 1998) or 80% (9/11; Davis et al., 2014) of HD cases. Due to the growing evidence of the presence of Tau pathology in HD patients and in an attempt to understand how it may or may not contribute to the pathology of the disease, different studies in which animal models and patients are used have tried to systematically analyze this question.

### mHtt and Tau Co-localization

Different approaches have been attempted to study if mHtt and Tau are directly or indirectly related to understand the mechanism of the confluence of both proteinopathies in HD patients. Some co-localization between mHtt aggregates (evidenced with EM48 antibody) and Tau deposits stained with antibodies that recognize 3R-Tau, 4R-Tau or pathologically phosphorylated Tau (AT-8 and pS199) has been detected in cortical and striatal sections of HD patients (Vuono et al., 2015). In contrast, other studies have failed to detect Tau within HD inclusions. For instance, Tau (Ht-7 antibody) could not be found in mHtt inclusions (evidenced with ubiquitin antibody) by immunofluorescence in cortical tissue of HD patients (Fernández-Nogales et al., 2014). Similarly, in animal models, no co-localization of both proteins could be found by confocal immunofluorescence using Tau pSer396 and mHtt (2B4 or EM48) antibodies in KI140 mice (Blum et al., 2015).

To clarify if there is or not an authentic co-localization between both proteins, different co-immunoprecipitation studies have been performed. In human tissue, no co-immunoprecipitation between both proteins was observed using the Tau 5 antibody that detects total Tau and the EM-48 in striatal homogenates of HD patients (Fernández-Nogales et al., 2014). No co-immunoprecipitation between both proteins was achieved either with cortical samples of KI140 mice nor was Tau detected in cortical Sarkosyl-insoluble protein fractions from R6/2 mice (Blum et al., 2015). Interestingly, in BIFC experiments *in vitro* with constructs with 25Q (wt) or 103Q mHtt and Tau fused with non-fluorescence halves of a fluorescence reporter protein, Blum et al. (2015) observed that when they put together 103Q and Tau, 103QHtt is recruited in the microtubular cytoskeleton network. Besides, Tau is hyperphosphorylated and, although its subcellular distribution is altered and its aggregation favored, it is not obvious the existence of a toxic interaction of both proteins and further studies are needed to clarify that.

### Tau-Positive Cytoplasmic Aggregates

In view of the abnormal Tau “processing” that we initially detected in HD brains (Fernández-Nogales et al., 2014) that leads to an alteration of 4R-Tau/3R-Tau ratio in favor of 4R-Tau isoforms (similar to that seen in some FTD forms caused by intronic Tau mutations), we explored the possibility of Tau deposits in HD brains. We detected granular cytoplasmic Tau deposits which often form perinuclear rings in cortical and striatal neurons (Fernández-Nogales et al., 2014) by immunohistochemistry with antibodies that recognize 4R-Tau isoforms (RD4), 3R-Tau isoforms (RD-3) or Total Tau (Tau-5 and HT-7), but not with anti-phospho-Tau antibodies. Vuono et al. (2015) detected similar Tau deposits like perinuclear rings, flame-shaped and globular inclusions as well as astrocytic plaques in striatum and cortex of HD subjects but, in this case, with an antibody against phosphorylated Tau (AT-8). Similarly, in a more recent study, Cisbani et al. (2017) also detected AT-8 positive neuronal inclusions—apart from NFTs and NTs—in striatum and cortex of HD patients.

### Tau-Positive Nuclear Membrane Invaginations

**Figure 1:**
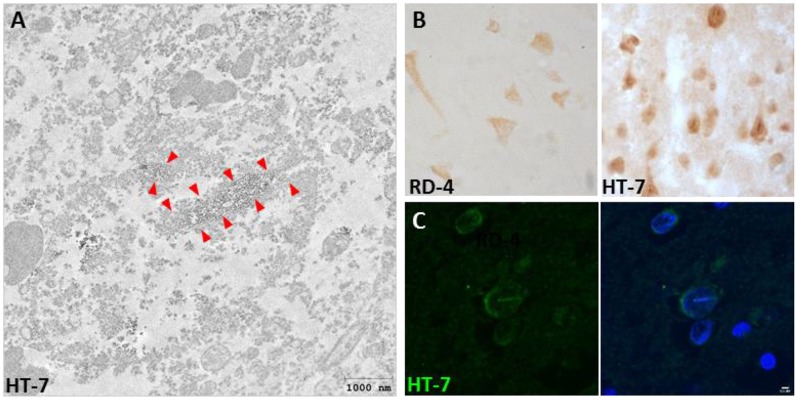
Presence of Tau nuclear indentations (TNIs) in Huntington’s disease (HD) brains. **(A)** Immunoelectron microscopy analysis of HD neurons with HT-7 positive nuclear indentations. Red arrows indicate the diaminobenzidine precipitate. **(B)** Immunohistochemistry with RD4 and Ht-7 antibodies in neurons of HD brain. **(C)** HT-7 immunofluorescence (green) with DAPI (blue) counterstaining in HD striatal neurons.

Interestingly, we also described for the first time the presence of Tau nuclear indentations (TNIs) also known as Tau Nuclear Rods (TNRs) in the striatum and cortex of HD patients (Fernández-Nogales et al., 2014). We detected TNIs using antibodies that recognize 4R-Tau isoforms (RD4), 3R-Tau isoforms (RD-3), Total Tau (Tau-5 and HT-7) or Tau oligomers (T22), but not with antibodies against phosphorylated Tau (such as AT-8 or PHF-1; Fernández-Nogales et al., 2014). Immuno-electron microscopy with HT-7 antibody revealed that this structure has an ordered filamentous ultrastructure immunopositive for Tau that fills neuronal invaginations of the nuclear envelope that partially or totally span the neuronal nuclear space (Fernández-Nogales et al., 2014). This new Tau histopathological hallmark thus seems to fill the previously reported neuronal nuclear membrane invaginations detected in ultrastructural analyses and whose incidence is higher in striatum of HD patients than in control subjects (Bots and Bruyn, 1981; Roos and Bots, 1983). Examples of TNIs evidenced by IHC, IF and immuno-EM are shown in Figure 1. The presence of TNIs in HD brains was confirmed in an independent study with a higher number of HD patient samples (Vuono et al., 2015) although, in this case, using the AT-8 antibody against phosphorylated Tau. In contrast, a recent study on samples from seven HD cases (Vonsattel grades 3 and 4), failed to detect TNIs (Baskota et al., 2019) and they reasoned that this may be due to technical features because the variety of antibodies they used did not detect neurons with cytoplasmic Tau staining, which are the ones displaying TNIs in the above-mentioned studies which do detect them. In HD mouse models, TNIs have also has been detected with Tau-5 and RD4 antibodies in the R6/1 and HD94 mice although with much lower abundance than in human HD tissue (Fernández-Nogales et al., 2014).

Interestingly, TNIs can be found in brain tissue of other neurodegenerative diseases. More precisely, in two Tauopathies: AD and FTD. Thus, we detected TNIs in the hippocampus of different Braak stage AD patients using the RD4 antibody (Fernández-Nogales et al., 2014). More recently, TNIs have also been detected in frontal and temporal cortex samples from two independent cohorts of patients with FTD-MAPT due to the MAPT intronic IVS10+16 mutation (Paonessa et al., 2019). Such mutation increases exon 10 inclusion and, therefore, increases the 4R-Tau/3R-Tau ratio in favor of 4R-Tau isoforms (similar to what we have described in HD brains).

Paonessa et al. (2019) also detected increased incidence of TNIs in IPSC-neurons derived from FTD-MAPT cases due to the MAPT IVS10+16 mutation and also, but to a lower extent, from FTD-MAPT cases due to missense P301L mutation that produces an aggregation-prone form of Tau.

Regarding the possible pathogenic relevance of TNIs, lamin dysfunction and neuronal nuclear indentations in AD have been linked to the improper cytoskeletal/nucleoskeletal coupling that was suggested as a novel mediator of neurotoxicity in Tauopathies (Frost et al., 2016) and more recently, pathological Tau has been shown to impair nucleocytoplasmic transport in Tau-overexpressing mice and AD brain tissue (Eftekharzadeh et al., 2018) which has been further confirmed recently (Paonessa et al., 2019). In this regard, nuclear integrity and nucleocytoplasmic transport have also been reported altered in Huntington‘s disease (Gasset-Rosa et al., 2017; Grima et al., 2017).

Regarding the mechanism by which TNIs get formed, analysis of a transgenic mouse model that overexpresses human 4R-Tau with a FTLD with the P301S Tau point mutation revealed that Tau alteration is sufficient for TNI formation/detection (Fernández-Nogales et al., 2017). Similarly, a *Drosophila* model with pan-neuronal expression of a disease-causing mutant form of human Tau, Tau^R406W^, produces nuclear invagination that co-localizes with phosphorylated Tau (Cornelison et al., 2019). These observations raised the question of whether increased Tau (either total Tau or 4R-Tau regardless of point mutations) fills nuclear invaginations formed by Tau-independent mechanisms or whether the incidence of nuclear envelope invaginations increases upon Tau alteration, for instance, because it stabilizes microtubule bundles that deform the nucleus. The latter is precisely what Paonessa et al. (2019) demonstrate in human iPSC-derived neurons with MAPT P301L and MAPT IVS16+ 10 mutations as treatment with nocodazole significantly reduced the proportion of neurons with nuclear invaginations and restored round nuclear morphology. This is in contrast with the fact that the number of nuclear indentations in hippocampal neurons of P301S mice is not higher than in wild type mice (Fernández-Nogales et al., 2017). In case the induction of nuclear envelope invagination upon Tau alteration occurred only in culture, TNI detection in histopathological analysis still emerges as an efficient way to screen for brains with altered Tau (levels, isoform ratio, or simply function). Additional work will be needed to clarify this.

## Pathogenic and Therapeutic Implications: Future Directions

Here we have shown multiple alterations of Tau in HD brain tissue which range from increased total levels, imbalance of alternative splicing-generated isoforms, hyperphosphorylation and truncation, to the formation of Tau-positive cytoplasmic aggregates and nuclear envelope invaginations. The likely pathogenic role of the latter—through interference of nuclear envelope function—and the possible mechanisms by which TNIs are formed are also discussed. A key question is whether correction of any of these tau alterations and of this Tau histopathological hallmark might have positive therapeutic implications for the disease.

Regarding the therapeutic implications of the current knowledge of the involvement of Tau in HD pathogenesis, a convincing evidence supporting that Tau contributes to HD pathogenesis originates from the beneficial effect of decreasing Tau expression in HD mouse models by combining with Tau knock-out mice—even partial reduction in heterozygous knock-out background (Fernández-Nogales et al., 2014). This beneficial effect might be due to the attenuation of the excess total levels of Tau in the cortex of HD mice or by attenuation specifically of the excess 4R-Tau observed both in striatum and cortex. But in any case, since gene silencing drugs are under development to attenuate both HTT expression for HD and to attenuate MAPT expression for FTD-MAPT (Mullard, 2019), it is conceivable that the combined therapy in HD patients might be more effective than HTT silencing alone.

## Ethics Statement

Brain specimens reviewed in this study from frontal cortex and striatum of HD subjects and controls were provided by Institute of Neuropathology (HUB-ICO-IDIBELL) Brain Bank (Hospitalet de Llobregat, Spain), the Neurological Tissue Bank of the IDIBAPS Biobank (Barcelona, Spain), the Banco de Tejidos Fundación Cien (BT-CIEN, Madrid, Spain) and the Netherlands Brain Bank (Amsterdam, The Netherlands). Written informed consent for brain removal after death for diagnostic and research purposes was obtained from brain donors and/or next of kin. Procedures, information and consent forms have been approved by the Bioethics Subcommittee of Centro Superior de Investigaciones Científicas (Madrid, Spain).

## Author Contributions

Both authors (MF-N and JL) revised the literature and wrote the manuscript.

## Conflict of Interest

The authors declare that the research was conducted in the absence of any commercial or financial relationships that could be construed as a potential conflict of interest.

## References

[B1] Andreadis A. (2005). Tau gene alternative splicing: expression patterns, regulation and modulation of function in normal brain and neurodegenerative diseases. Biochim. Biophys. Acta 1739, 91–103. 10.1016/j.bbadis.2004.08.010 15615629

[B2] Andreadis A. Broderick J. A. Kosik K. S. (1995). Relative exon affinities and suboptimal splice site signals lead to non-equivalence of two cassette exons. Nucleic Acids Res. 23, 3585–3593. 10.1093/nar/23.17.3585 7567473 PMC307241

[B3] Andreadis A. Brown W. M. Kosik K. S. (1992). Structure and novel exons of the human tau gene. Biochemistry 31, 10626–10633. 10.1021/bi00158a027 1420178

[B4] Andrew S. E. Goldberg Y. P. Kremer B. Telenius H. Theilmann J. Adam S.. (1993). The relationship between trinucleotide (CAG) repeat length and clinical features of Huntington’s disease. Nat. Genet. 4, 398–403. 10.1038/ng0893-398 8401589

[B5] Arendt T. Stieler J. T. Holzer M. (2016). Tau and tauopathies. Brain Res. Bull 126, 238–292. 10.1016/j.brainresbull.2016.08.018 27615390

[B6] Barghorn S. Zheng-Fischhofer Q. Ackmann M. Biernat J. von Bergen M. Mandelkow E. M.. (2000). Structure, microtubule interactions, and paired helical filament aggregation by tau mutants of frontotemporal dementias. Biochemistry 39, 11714–11721. 10.1021/bi000850r 10995239

[B7] Baskota S. U. Lopez O. L. Greenamyre J. T. Kofler J. (2019). Spectrum of tau pathologies in Huntington’s disease. Lab. Invest. 99, 1068–1077. 10.1038/s41374-018-0166-9 30573872 PMC9342691

[B8] Bates G. P. Dorsey R. Gusella J. F. Hayden M. R. Kay C. Leavitt B. R.. (2015). Huntington disease. Nat. Rev. Dis. Primers 1:15005. 10.1038/nrdp.2015.5 27188817

[B9] Beaulieu J. M. Sotnikova T. D. Yao W. D. Kockeritz L. Woodgett J. R. Gainetdinov R. R.. (2004). Lithium antagonizes dopamine-dependent behaviors mediated by an AKT/glycogen synthase kinase 3 signaling cascade. Proc. Natl. Acad. Sci. U S A 101, 5099–5104. 10.1073/pnas.0307921101 15044694 PMC387380

[B10] Benhelli-Mokrani H. Mansuroglu Z. Chauderlier A. Albaud B. Gentien D. Sommer S.. (2018). Genome-wide identification of genic and intergenic neuronal DNA regions bound by Tau protein under physiological and stress conditions. Nucleic Acids Res. 46, 11405–11422. 10.1093/nar/gky929 30321409 PMC6265482

[B11] Blum D. Herrera F. Francelle L. Mendes T. Basquin M. Obriot H.. (2015). Mutant huntingtin alters Tau phosphorylation and subcellular distribution. Hum. Mol. Genet. 24, 76–85. 10.1093/hmg/ddu421 25143394

[B12] Bots G. T. Bruyn G. W. (1981). Neuropathological changes of the nucleus accumbens in Huntington’s chorea. Acta Neuropathol. 55, 21–22. 10.1007/bf00691525 6215820

[B13] Cabrera J. R. Lucas J. J. (2017). MAP2 splicing is altered in Huntington’s disease. Brain Pathol. 27, 181–189. 10.1111/bpa.12387 27098187 PMC8029395

[B14] Caceres A. Kosik K. S. (1990). Inhibition of neurite polarity by tau antisense oligonucleotides in primary cerebellar neurons. Nature 343, 461–463. 10.1038/343461a0 2105469

[B15] Caillet-Boudin M. L. Buee L. Sergeant N. Lefebvre B. (2015). Regulation of human MAPT gene expression. Mol. Neurodegener. 10:28. 10.1186/s13024-015-0025-8 26170022 PMC4499907

[B16] Caparros-Lefebvre D. Kerdraon O. Devos D. Dhaenens C. M. Blum D. Maurage C. A.. (2009). Association of corticobasal degeneration and Huntington’s disease: can Tau aggregates protect Huntingtin toxicity? Mov. Disord. 24, 1089–1090. 10.1002/mds.22204 19243029

[B17] Chen J. Kanai Y. Cowan N. J. Hirokawa N. (1992). Projection domains of MAP2 and tau determine spacings between microtubules in dendrites and axons. Nature 360, 674–677. 10.1038/360674a0 1465130

[B18] Cisbani G. Maxan A. Kordower J. H. Planel E. Freeman T. B. Cicchetti F. (2017). Presence of tau pathology within foetal neural allografts in patients with Huntington’s and Parkinson’s disease. Brain 140, 2982–2992. 10.1093/brain/awx255 29069396 PMC5841208

[B19] Cleveland D. W. Hwo S. Y. Kirschner M. W. (1977). Purification of tau, a microtubule-associated protein that induces assembly of microtubules from purified tubulin. J. Mol. Biol. 116, 207–225. 10.1016/0022-2836(77)90213-3 599557

[B20] Cohen T. J. Guo J. L. Hurtado D. E. Kwong L. K. Mills I. P. Trojanowski J. Q.. (2011). The acetylation of tau inhibits its function and promotes pathological tau aggregation. Nat. Commun. 2:252. 10.1038/ncomms1255 21427723 PMC3120096

[B22] Cornelison G. L. Levy S. A. Jenson T. Frost B. (2019). Tau-induced nuclear envelope invagination causes a toxic accumulation of mRNA in *Drosophila*. Aging Cell 18:e12847. 10.1111/acel.12847 30411463 PMC6351838

[B23] Corrochano S. Renna M. Carter S. Chrobot N. Kent R. Stewart M.. (2012). α-Synuclein levels modulate Huntington’s disease in mice. Hum. Mol. Genet. 21, 485–494. 10.1093/hmg/ddr477 22010050 PMC3259010

[B24] Coudert L. Nonaka T. Bernard E. Hasegawa M. Schaeffer L. Leblanc P. (2019). Phosphorylated and aggregated TDP-43 with seeding properties are induced upon mutant Huntingtin (mHtt) polyglutamine expression in human cellular models. Cell. Mol. Life Sci. 76, 2615–2632. 10.1007/s00018-019-03059-8 30863908 PMC11105362

[B25] Davies S. W. Turmaine M. Cozens B. A. DiFiglia M. Sharp A. H. Ross C. A.. (1997). Formation of neuronal intranuclear inclusions underlies the neurological dysfunction in mice transgenic for the HD mutation. Cell 90, 537–548. 10.1016/s0092-8674(00)80513-9 9267033

[B26] Davis M. Y. Keene C. D. Jayadev S. Bird T. (2014). The co-occurrence of Alzheimer’s disease and Huntington’s disease: a neuropathological study of 15 elderly Huntington’s disease subjects. J. Huntingtons Dis. 3, 209–217. 10.3233/jhd-140111 25062863

[B27] Deckel A. W. Elder R. Fuhrer G. (2002). Biphasic developmental changes in Ca2+/calmodulin-dependent proteins in R6/2 Huntington’s disease mice. Neuroreport 13, 707–711. 10.1097/00001756-200204160-00034 11973475

[B28] Derisbourg M. Leghay C. Chiappetta G. Fernandez-Gomez F. J. Laurent C. Demeyer D.. (2015). Role of the Tau N-terminal region in microtubule stabilization revealed by new endogenous truncated forms. Sci. Rep. 5:9659. 10.1038/srep09659 25974414 PMC4431475

[B29] DiFiglia M. Sapp E. Chase K. O. Davies S. W. Bates G. P. Vonsattel J. P.. (1997). Aggregation of huntingtin in neuronal intranuclear inclusions and dystrophic neurites in brain. Science 277, 1990–1993. 10.1126/science.277.5334.1990 9302293

[B30] Dixit R. Ross J. L. Goldman Y. E. Holzbaur E. L. (2008). Differential regulation of dynein and kinesin motor proteins by tau. Science 319, 1086–1089. 10.1126/science.1152993 18202255 PMC2866193

[B31] Eftekharzadeh B. Daigle J. G. Kapinos L. E. Coyne A. Schiantarelli J. Carlomagno Y.. (2018). Tau protein disrupts nucleocytoplasmic transport in Alzheimer’s disease. Neuron 99, 925.e7–940.e7. 10.1016/j.neuron.2018.07.039 30189209 PMC6240334

[B32] Fernández-Nogales M. Cabrera J. R. Santos-Galindo M. Hoozemans J. J. Ferrer I. Rozemuller A. J.. (2014). Huntington’s disease is a four-repeat tauopathy with tau nuclear rods. Nat. Med. 20, 881–885. 10.1038/nm.3617 25038828

[B33] Fernández-Nogales M. Hernández F. Miguez A. Alberch J. Ginés S. Peréz-Navarro E.. (2015). Decreased glycogen synthase kinase-3 levels and activity contribute to Huntington’s disease. Hum. Mol. Genet. 24, 5040–5052. 10.1093/hmg/ddv224 26082469

[B34] Fernández-Nogales M. Santos-Galindo M. Merchán-Rubira J. Hoozemans J. J. M. Rábano A. Ferrer I.. (2017). Tau-positive nuclear indentations in P301S tauopathy mice. Brain Pathol. 27, 314–322. 10.1111/bpa.12407 27338164 PMC8029483

[B35] Frost B. Bardai F. H. Feany M. B. (2016). Lamin dysfunction mediates neurodegeneration in tauopathies. Curr. Biol. 26, 129–136. 10.1016/j.cub.2015.11.039 26725200 PMC4713335

[B36] Fujioka Y. Ishigaki S. Masuda A. Iguchi Y. Udagawa T. Watanabe H.. (2013). FUS-regulated region- and cell-type-specific transcriptome is associated with cell selectivity in ALS/FTLD. Sci. Rep. 3:2388. 10.1038/srep02388 23925123 PMC3737506

[B37] Gasset-Rosa F. Chillon-Marinas C. Goginashvili A. Atwal R. S. Artates J. W. Tabet R.. (2017). Polyglutamine-expanded huntingtin exacerbates age-related disruption of nuclear integrity and nucleocytoplasmic transport. Neuron 94, 48.e4–57.e4. 10.1016/j.neuron.2017.03.027 28384474 PMC5479704

[B21] Genetic Modifiers of Huntington’s Disease (GeM-HD) Consortium. (2019). CAG repeat not polyglutamine length determines timing of Huntington’s disease onset. Cell 178, 887.e14–900.e14. 10.1016/j.cell.2019.06.036 31398342 PMC6700281

[B39] Goedert M. Jakes R. (2005). Mutations causing neurodegenerative tauopathies. Biochim. Biophys. Acta 1739, 240–250. 10.1016/j.bbadis.2004.08.007 15615642

[B40] Goedert M. Spillantini M. G. (2011). Pathogenesis of the tauopathies. J. Mol. Neurosci. 45, 425–431. 10.1007/s12031-011-9593-4 21785996

[B41] Goedert M. Spillantini M. G. Jakes R. Crowther R. A. Vanmechelen E. Probst A.. (1995). Molecular dissection of the paired helical filament. Neurobiol. Aging 16, 325–334. 10.1016/0197-4580(95)00017-9 7566342

[B42] Goedert M. Spillantini M. G. Jakes R. Rutherford D. Crowther R. A. (1989). Multiple isoforms of human microtubule-associated protein tau: sequences and localization in neurofibrillary tangles of Alzheimer’s disease. Neuron 3, 519–526. 10.1016/0896-6273(89)90210-9 2484340

[B43] Gong C. X. Liu F. Wu G. Rossie S. Wegiel J. Li L.. (2004). Dephosphorylation of microtubule-associated protein tau by protein phosphatase 5. J. Neurochem. 88, 298–310. 10.1111/j.1471-4159.2004.02147.x 14690518

[B44] Graham R. K. Deng Y. Slow E. J. Haigh B. Bissada N. Lu G.. (2006). Cleavage at the caspase-6 site is required for neuronal dysfunction and degeneration due to mutant huntingtin. Cell 125, 1179–1191. 10.1016/j.cell.2006.04.026 16777606

[B45] Gratuze M. Noël A. Julien C. Cisbani G. Milot-Rousseau P. Morin F.. (2015). Tau hyperphosphorylation and deregulation of calcineurin in mouse models of Huntington’s disease. Hum. Mol. Genet. 24, 86–99. 10.1093/hmg/ddu456 25205109

[B46] Grima J. C. Daigle J. G. Arbez N. Cunningham K. C. Zhang K. Ochaba J.. (2017). Mutant huntingtin disrupts the nuclear pore complex. Neuron 94, 93.e6–107.e6. 10.1016/j.neuron.2017.03.023 28384479 PMC5595097

[B47] Gusella J. F. MacDonald M. E. Ambrose C. M. Duyao M. P. (1993). Molecular genetics of Huntington’s disease. Arch. Neurol. 50, 1157–1163. 10.1001/archneur.1993.00540110037003 8215974

[B49] Hedreen J. C. Folstein S. E. (1995). Early loss of neostriatal striosome neurons in Huntington’s disease. J. Neuropathol. Exp. Neurol. 54, 105–120. 10.1097/00005072-199501000-00013 7815073

[B200] Hernández-Ortega K. Garcia-Esparcia P. Gil L. Lucas J. J. Ferrer I. (2016). Altered machinery of protein synthesis in Alzheimer’s: from the nucleolus to the ribosome. Brain Pathol. 26, 593–605. 10.1111/bpa.12335 26512942 PMC8029302

[B50] Hernandez F. Lucas J. J. Avila J. (2013). GSK3 and tau: two convergence points in Alzheimer’s disease. J. Alzheimers Dis. 33, S141–S144. 10.3233/jad-2012-129025 22710914

[B51] Herrera F. Outeiro T. F. (2012). α-synuclein modifies huntingtin aggregation in living cells. FEBS Lett. 586, 7–12. 10.1016/j.febslet.2011.11.019 22119730

[B52] Houlden H. Baker M. Morris H. R. MacDonald N. Pickering-Brown S. Adamson J.. (2001). Corticobasal degeneration and progressive supranuclear palsy share a common tau haplotype. Neurology 56, 1702–1706. 10.1212/wnl.56.12.1702 11425937

[B53] Hutton M. Lendon C. L. Rizzu P. Baker M. Froelich S. Houlden H.. (1998). Association of missense and 5’-splice-site mutations in tau with the inherited dementia FTDP-17. Nature 393, 702–705. 10.1038/31508 9641683

[B54] Ishigaki S. Fujioka Y. Okada Y. Riku Y. Udagawa T. Honda D.. (2017). Altered tau isoform ratio caused by loss of FUS and SFPQ function leads to FTLD-like phenotypes. Cell Rep. 18, 1118–1131. 10.1016/j.celrep.2017.01.013 28147269

[B55] Jellinger K. A. (1998). Alzheimer-type lesions in Huntington’s disease. J. Neural Transm. 105, 787–799. 10.1007/s007020050095 9869319

[B57] Kadavath H. Hofele R. V. Biernat J. Kumar S. Tepper K. Urlaub H.. (2015). Tau stabilizes microtubules by binding at the interface between tubulin heterodimers. Proc. Natl. Acad. Sci. U S A 112, 7501–7506. 10.1073/pnas.1504081112 26034266 PMC4475932

[B58] Kaltenbach L. S. Romero E. Becklin R. R. Chettier R. Bell R. Phansalkar A.. (2007). Huntingtin interacting proteins are genetic modifiers of neurodegeneration. PLoS Genet. 3:e82. 10.1371/journal.pgen.0030082 17500595 PMC1866352

[B59] Kempf M. Clement A. Faissner A. Lee G. Brandt R. (1996). Tau binds to the distal axon early in development of polarity in a microtubule- and microfilament-dependent manner. J. Neurosci. 16, 5583–5592. 10.1523/JNEUROSCI.16-18-05583.1996 8795614 PMC6578978

[B60] Kim S. D. Fung V. S. (2014). An update on Huntington’s disease: from the gene to the clinic. Curr. Opin. Neurol. 27, 477–483. 10.1097/WCO.0000000000000116 24978638

[B61] Kosik K. S. Orecchio L. D. Bakalis S. Neve R. L. (1989). Developmentally regulated expression of specific tau sequences. Neuron 2, 1389–1397. 10.1016/0896-6273(89)90077-9 2560640

[B62] Ledesma M. D. Bonay P. Avila J. (1995). Tau protein from Alzheimer’s disease patients is glycated at its tubulin-binding domain. J. Neurochem. 65, 1658–1664. 10.1046/j.1471-4159.1995.65041658.x 7561862

[B63] L’Episcopo F. Drouin-Ouellet J. Tirolo C. Pulvirenti A. Giugno R. Testa N.. (2016). GSK-3β-induced Tau pathology drives hippocampal neuronal cell death in Huntington’s disease: involvement of astrocyte-neuron interactions. Cell Death Dis. 7:e2206. 10.1038/cddis.2016.104 27124580 PMC4855649

[B64] Li X. Liu M. Cai Z. Wang G. Li X. (2010). Regulation of glycogen synthase kinase-3 during bipolar mania treatment. Bipolar Disord. 12, 741–752. 10.1111/j.1399-5618.2010.00866.x 21040291 PMC3059222

[B65] Lim N. K. Hung L. W. Pang T. Y. McLean C. A. Liddell J. R. Hilton J. B.. (2014). Localized changes to glycogen synthase kinase-3 and collapsin response mediator protein-2 in the Huntington’s disease affected brain. Hum. Mol. Genet. 23, 4051–4063. 10.1093/hmg/ddu119 24634145

[B66] Liu F. Gong C. X. (2008). Tau exon 10 alternative splicing and tauopathies. Mol. Neurodegener. 3:8. 10.1186/1750-1326-3-8 18616804 PMC2483273

[B67] Liu P. Smith B. R. Huang E. S. Mahesh A. Vonsattel J. P. G. Petersen A. J.. (2019). A soluble truncated tau species related to cognitive dysfunction and caspase-2 is elevated in the brain of Huntington’s disease patients. Acta Neuropathol. Commun. 7:111. 10.1186/s40478-019-0764-9 31358058 PMC6664763

[B68] Lovestone S. Reynolds C. H. Latimer D. Davis D. R. Anderton B. H. Gallo J. M.. (1994). Alzheimer’s disease-like phosphorylation of the microtubule-associated protein tau by glycogen synthase kinase-3 in transfected mammalian cells. Curr. Biol. 4, 1077–1086. 10.1016/s0960-9822(00)00246-3 7704571

[B69] Lu M. Kosik K. S. (2001). Competition for microtubule-binding with dual expression of tau missense and splice isoforms. Mol. Biol. Cell 12, 171–184. 10.1091/mbc.12.1.171 11160831 PMC30576

[B48] MacDonald M. E. Ambrose C. M. Duyao M. P. Myers R. H. Lin C. Srinidhi L.. (1993). A novel gene containing a trinucleotide repeat that is expanded and unstable on Huntington’s disease chromosomes. Cell 72, 971–983. 10.1016/0092-8674(93)90585-e 8458085

[B70] Maina M. B. Bailey L. J. Wagih S. Biasetti L. Pollack S. J. Quinn J. P.. (2018). The involvement of tau in nucleolar transcription and the stress response. Acta Neuropathol. Commun. 6:70. 10.1186/s40478-018-0565-6 30064522 PMC6066928

[B71] Martí E. (2016). RNA toxicity induced by expanded CAG repeats in Huntington’s disease. Brain Pathol. 26, 779–786. 10.1111/bpa.12427 27529325 PMC8029335

[B72] Martin L. Latypova X. Terro F. (2011). Post-translational modifications of tau protein: implications for Alzheimer’s disease. Neurochem. Int. 58, 458–471. 10.1016/j.neuint.2010.12.023 21215781

[B73] Mayer A. Siegel N. R. Schwartz A. L. Ciechanover A. (1989). Degradation of proteins with acetylated amino termini by the ubiquitin system. Science 244, 1480–1483. 10.1126/science.2544030 2544030

[B74] McIntosh G. C. Jameson H. D. Markesbery W. R. (1978). Huntington disease associated with Alzheimer disease. Ann. Neurol. 3, 545–548. 10.1002/ana.410030616 150253

[B75] Min S. W. Cho S. H. Zhou Y. Schroeder S. Haroutunian V. Seeley W. W.. (2010). Acetylation of tau inhibits its degradation and contributes to tauopathy. Neuron 67, 953–966. 10.1016/j.neuron.2010.08.044 20869593 PMC3035103

[B76] Mitchell I. J. Cooper A. J. Griffiths M. R. (1999). The selective vulnerability of striatopallidal neurons. Prog. Neurobiol. 59, 691–719. 10.1016/s0301-0082(99)00019-2 10845758

[B77] Morishima-Kawashima M. Hasegawa M. Takio K. Suzuki M. Titani K. Ihara Y. (1993). Ubiquitin is conjugated with amino-terminally processed tau in paired helical filaments. Neuron 10, 1151–1160. 10.1016/0896-6273(93)90063-w 8391280

[B78] Moss R. J. Mastri A. R. Schut L. J. (1988). The coexistence and differentiation of late onset Huntington’s disease and Alzheimer’s disease. A case report and review of the literature. J. Am. Geriatr. Soc. 36, 237–241. 10.1111/j.1532-5415.1988.tb01807.x 2963060

[B79] Mullard A. (2019). Pioneering antisense drug heads into pivotal trials for Huntington disease. Nat. Rev. Drug Discov. 18, 161–163. 10.1038/d41573-019-00018-7 30824887

[B80] Myers A. J. Pittman A. M. Zhao A. S. Rohrer K. Kaleem M. Marlowe L.. (2007). The MAPT H1c risk haplotype is associated with increased expression of tau and especially of 4 repeat containing transcripts. Neurobiol. Dis. 25, 561–570. 10.1016/j.nbd.2006.10.018 17174556

[B81] Myers R. H. Sax D. S. Schoenfeld M. Bird E. D. Wolf P. A. Vonsattel J. P.. (1985). Late onset of Huntington’s disease. J. Neurol. Neurosurg. Psychiatry 48, 530–534. 10.1136/jnnp.48.6.530 3159849 PMC1028368

[B82] Nalavade R. Griesche N. Ryan D. P. Hildebrand S. Krauss S. (2013). Mechanisms of RNA-induced toxicity in CAG repeat disorders. Cell Death Dis. 4:e752. 10.1038/cddis.2013.276 23907466 PMC3763438

[B83] Naro C. Sette C. (2013). Phosphorylation-mediated regulation of alternative splicing in cancer. Int. J. Cell Biol. 2013:151839. 10.1155/2013/151839 24069033 PMC3771450

[B84] Nasir J. Floresco S. B. O’Kusky J. R. Diewert V. M. Richman J. M. Zeisler J.. (1995). Targeted disruption of the Huntington’s disease gene results in embryonic lethality and behavioral and morphological changes in heterozygotes. Cell 81, 811–823. 10.1016/0092-8674(95)90542-1 7774020

[B85] Neve R. L. Harris P. Kosik K. S. Kurnit D. M. Donlon T. A. (1986). Identification of cDNA clones for the human microtubule-associated protein tau and chromosomal localization of the genes for tau and microtubule-associated protein 2. Brain Res. 387, 271–280. 10.1016/0169-328x(86)90033-1 3103857

[B86] Panda D. Samuel J. C. Massie M. Feinstein S. C. Wilson L. (2003). Differential regulation of microtubule dynamics by three- and four-repeat tau: implications for the onset of neurodegenerative disease. Proc. Natl. Acad. Sci. U S A 100, 9548–9553. 10.1073/pnas.1633508100 12886013 PMC170955

[B87] Paonessa F. Evans L. D. Solanki R. Larrieu D. Wray S. Hardy J.. (2019). Microtubules deform the nuclear membrane and disrupt nucleocytoplasmic transport in tau-mediated frontotemporal dementia. Cell Rep. 26, 582.e585–593.e585. 10.1016/j.celrep.2018.12.085 30650353 PMC6335264

[B88] Park S. A. Ahn S. I. Gallo J. M. (2016). Tau mis-splicing in the pathogenesis of neurodegenerative disorders. BMB Rep. 49, 405–413. 10.5483/bmbrep.2016.49.8.084 27222125 PMC5070727

[B89] Pei J. J. Tanaka T. Tung Y. C. Braak E. Iqbal K. Grundke-Iqbal I. (1997). Distribution, levels and activity of glycogen synthase kinase-3 in the Alzheimer disease brain. J. Neuropathol. Exp. Neurol. 56, 70–78. 10.1097/00005072-199701000-00007 8990130

[B90] Pittman A. M. Myers A. J. Duckworth J. Bryden L. Hanson M. Abou-Sleiman P.. (2004). The structure of the tau haplotype in controls and in progressive supranuclear palsy. Hum. Mol. Genet. 13, 1267–1274. 10.1093/hmg/ddh138 15115761

[B91] Poorkaj P. Bird T. D. Wijsman E. Nemens E. Garruto R. M. Anderson L.. (1998). Tau is a candidate gene for chromosome 17 frontotemporal dementia. Ann. Neurol. 43, 815–825. 10.1002/ana.410430617 9629852

[B92] Qian W. Liu F. (2014). Regulation of alternative splicing of tau exon 10. Neurosci. Bull. 30, 367–377. 10.1007/s12264-013-1411-2 24627328 PMC5562657

[B93] Reyes M. G. Gibbons S. (1985). Dementia of the Alzheimer’s type and Huntington’s disease. Neurology 35, 273–277. 10.1212/wnl.35.2.273 3155827

[B94] Roos R. A. Bots G. T. (1983). Nuclear membrane indentations in Huntington’s chorea. J. Neurol. Sci. 61, 37–47. 10.1016/0022-510x(83)90053-9 6226764

[B95] Saavedra A. Garcia-Martinez J. M. Xifro X. Giralt A. Torres-Peraza J. F. Canals J. M.. (2010). PH domain leucine-rich repeat protein phosphatase 1 contributes to maintain the activation of the PI3K/Akt pro-survival pathway in Huntington’s disease striatum. Cell Death Differ. 17, 324–335. 10.1038/cdd.2009.127 19745829

[B96] Sapp E. Schwarz C. Chase K. Bhide P. G. Young A. B. Penney J.. (1997). Huntingtin localization in brains of normal and Huntington’s disease patients. Ann. Neurol. 42, 604–612. 10.1002/ana.410420411 9382472

[B97] Sathasivam K. Neueder A. Gipson T. A. Landles C. Benjamin A. C. Bondulich M. K.. (2013). Aberrant splicing of HTT generates the pathogenic exon 1 protein in Huntington disease. Proc. Natl. Acad. Sci. U S A 110, 2366–2370. 10.1073/pnas.1221891110 23341618 PMC3568346

[B98] Schilling J. Broemer M. Atanassov I. Duernberger Y. Vorberg I. Dieterich C.. (2019). Deregulated splicing is a major mechanism of RNA-induced toxicity in Huntington’s disease. J. Mol. Biol. 431, 1869–1877. 10.1016/j.jmb.2019.01.034 30711541

[B99] Schulte J. Littleton J. T. (2011). The biological function of the Huntingtin protein and its relevance to Huntington’s disease pathology. Curr. Trends Neurol. 5, 65–78. 22180703 PMC3237673

[B100] Schwab C. Arai T. Hasegawa M. Yu S. McGeer P. L. (2008). Colocalization of transactivation-responsive DNA-binding protein 43 and huntingtin in inclusions of Huntington disease. J. Neuropathol. Exp. Neurol. 67, 1159–1165. 10.1097/nen.0b013e31818e8951 19018245

[B101] Sergeant N. Bretteville A. Hamdane M. Caillet-Boudin M. L. Grognet P. Bombois S.. (2008). Biochemistry of Tau in Alzheimer’s disease and related neurological disorders. Expert Rev. Proteomics 5, 207–224. 10.1586/14789450.5.2.207 18466052

[B102] Sergeant N. Delacourte A. Buée L. (2005). Tau protein as a differential biomarker of tauopathies. Biochim. Biophys. Acta 1739, 179–197. 10.1016/j.bbadis.2004.06.020 15615637

[B103] Shieh S. Y. Bonini N. M. (2011). Genes and pathways affected by CAG-repeat RNA-based toxicity in *Drosophila*. Hum. Mol. Genet. 20, 4810–4821. 10.1093/hmg/ddr420 21933837 PMC3221540

[B104] Siano G. Varisco M. Caiazza M. C. Quercioli V. Mainardi M. Ippolito C.. (2019). Tau modulates VGluT1 expression. J. Mol. Biol. 431, 873–884. 10.1016/j.jmb.2019.01.023 30664870

[B105] Smith-Dijak A. I. Sepers M. D. Raymond L. A. (2019). Alterations in synaptic function and plasticity in Huntington disease. J. Neurochem. 150, 346–365. 10.1111/jnc.14723 31095731

[B106] Snell R. G. MacMillan J. C. Cheadle J. P. Fenton I. Lazarou L. P. Davies P.. (1993). Relationship between trinucleotide repeat expansion and phenotypic variation in Huntington’s disease. Nat. Genet. 4, 393–397. 10.1038/ng0893-393 8401588

[B108] Spillantini M. G. Bird T. D. Ghetti B. (1998). Frontotemporal dementia and Parkinsonism linked to chromosome 17: a new group of tauopathies. Brain Pathol. 8, 387–402. 10.1111/j.1750-3639.1998.tb00162.x 9546295 PMC8098460

[B107] Spillantini M. G. Goedert M. (2013). Tau pathology and neurodegeneration. Lancet Neurol. 12, 609–622. 10.1016/S1474-4422(13)70090-5 23684085

[B109] Stamer K. Vogel R. Thies E. Mandelkow E. Mandelkow E. M. (2002). Tau blocks traffic of organelles, neurofilaments and APP vesicles in neurons and enhances oxidative stress. J. Cell Biol. 156, 1051–1063. 10.1083/jcb.200108057 11901170 PMC2173473

[B110] St-Amour I. Turgeon A. Goupil C. Planel E. Hebert S. S. (2018). Co-occurrence of mixed proteinopathies in late-stage Huntington’s disease. Acta Neuropathol. 135, 249–265. 10.1007/s00401-017-1786-7 29134321

[B111] Stefansson H. Helgason A. Thorleifsson G. Steinthorsdottir V. Masson G. Barnard J.. (2005). A common inversion under selection in Europeans. Nat. Genet. 37, 129–137. 10.1038/ng1508 15654335

[B112] Sturrock A. Leavitt B. R. (2010). The clinical and genetic features of Huntington disease. J. Geriatr. Psychiatry Neurol. 23, 243–259. 10.1177/0891988710383573 20923757

[B113] Takuma H. Arawaka S. Mori H. (2003). Isoforms changes of tau protein during development in various species. Brain Res. Dev. Brain Res. 142, 121–127. 10.1016/s0165-3806(03)00056-7 12711363

[B114] Tomas-Zapico C. Diez-Zaera M. Ferrer I. Gomez-Ramos P. Moran M. A. Miras-Portugal M. T.. (2012). α-Synuclein accumulates in huntingtin inclusions but forms independent filaments and its deficiency attenuates early phenotype in a mouse model of Huntington’s disease. Hum. Mol. Genet. 21, 495–510. 10.1093/hmg/ddr507 22045698

[B115] Tracy T. E. Sohn P. D. Minami S. S. Wang C. Min S. W. Li Y.. (2016). Acetylated tau obstructs kibra-mediated signaling in synaptic plasticity and promotes tauopathy-related memory loss. Neuron 90, 245–260. 10.1016/j.neuron.2016.03.005 27041503 PMC4859346

[B116] Trinczek B. Ebneth A. Mandelkow E. M. Mandelkow E. (1999). Tau regulates the attachment/detachment but not the speed of motors in microtubule-dependent transport of single vesicles and organelles. J. Cell Sci. 112, 2355–2367. 10381391 10.1242/jcs.112.14.2355

[B117] Violet M. Delattre L. Tardivel M. Sultan A. Chauderlier A. Caillierez R.. (2014). A major role for Tau in neuronal DNA and RNA protection *in vivo* under physiological and hyperthermic conditions. Front. Cell. Neurosci. 8:84. 10.3389/fncel.2014.00084 24672431 PMC3957276

[B118] Vonsattel J. P. DiFiglia M. (1998). Huntington disease. J. Neuropathol. Exp. Neurol. 57, 369–384. 10.1097/00005072-199805000-00001 9596408

[B119] Vonsattel J. P. Del Amaya M. P. Keller C. E. (2008). Twenty-first century brain banking. Processing brains for research: the Columbia University methods. Acta Neuropathol. 115, 509–532. 10.1007/s00401-007-0311-9 17985145 PMC2292479

[B120] Vuono R. Winder-Rhodes S. de Silva R. Cisbani G. Drouin-Ouellet J. REGISTRY Investigators of the European Huntington’s Disease Network. (2015). The role of tau in the pathological process and clinical expression of Huntington’s disease. Brain 138, 1907–1918. 10.1093/brain/awv107 25953777 PMC4572485

[B121] Wang Y. Mandelkow E. (2016). Tau in physiology and pathology. Nat. Rev. Neurosci. 17, 5–21. 10.1038/nrn.2015.1 26631930

[B122] Warrick J. M. Paulson H. L. Gray-Board G. L. Bui Q. T. Fischbeck K. H. Pittman R. N.. (1998). Expanded polyglutamine protein forms nuclear inclusions and causes neural degeneration in *Drosophila*. Cell 93, 939–949. 10.1016/s0092-8674(00)81200-3 9635424

[B123] Wei Y. Qu M. H. Wang X. S. Chen L. Wang D. L. Liu Y.. (2008). Binding to the minor groove of the double-strand, tau protein prevents DNA from damage by peroxidation. PLoS One 3:e2600. 10.1371/journal.pone.0002600 18596978 PMC2432501

[B124] Weingarten M. D. Lockwood A. H. Hwo S. Y. Kirschner M. W. (1975). A protein factor essential for microtubule assembly. Proc. Natl. Acad. Sci. U S A 72, 1858–1862. 10.1073/pnas.72.5.1858 1057175 PMC432646

[B125] Wischik C. M. Novak M. Thogersen H. C. Edwards P. C. Runswick M. J. Jakes R.. (1988). Isolation of a fragment of tau derived from the core of the paired helical filament of Alzheimer disease. Proc. Natl. Acad. Sci. U S A 85, 4506–4510. 10.1073/pnas.85.12.4506 3132715 PMC280459

[B126] Witman G. B. Cleveland D. W. Weingarten M. D. Kirschner M. W. (1976). Tubulin requires tau for growth onto microtubule initiating sites. Proc. Natl. Acad. Sci. U S A 73, 4070–4074. 10.1073/pnas.73.11.4070 1069293 PMC431332

[B127] Yin X. Jin N. Gu J. Shi J. Zhou J. Gong C. X.. (2012). Dual-specificity tyrosine phosphorylation-regulated kinase 1A (Dyrk1A) modulates serine/arginine-rich protein 55 (SRp55)-promoted Tau exon 10 inclusion. J. Biol. Chem. 287, 30497–30506. 10.1074/jbc.m112.355412 22767602 PMC3436298

[B128] Zhang Z. Song M. Liu X. Kang S. S. Kwon I. S. Duong D. M.. (2014). Cleavage of tau by asparagine endopeptidase mediates the neurofibrillary pathology in Alzheimer’s disease. Nat. Med. 20, 1254–1262. 10.1038/nm.3700 25326800 PMC4224595

[B129] Zheng Z. Diamond M. I. (2012). Huntington disease and the huntingtin protein. Prog. Mol. Biol. Transl. Sci. 107, 189–214. 10.1016/B978-0-12-385883-2.00010-2 22482451

[B130] Zoghbi H. Y. Orr H. T. (2000). Glutamine repeats and neurodegeneration. Annu. Rev. Neurosci. 23, 217–247. 10.1146/annurev.neuro.23.1.217 10845064

[B131] Zuccato C. Cattaneo E. (2014). Huntington’s disease. Handb. Exp. Pharmacol. 220, 357–409. 10.1007/978-3-642-45106-5_14 24668480

